# Enhancement of Diffusion Assisted Bonding of the Bimetal Composite of Austenitic/Ferric Steels via Intrinsic Interlayers

**DOI:** 10.3390/ma14092416

**Published:** 2021-05-06

**Authors:** Chenwei He, Guangshan Pan, Lu Xie, Qing Peng

**Affiliations:** 1 Reactor Engineering and Safety Research Center, China Nuclear Power Technology Research Institute Co Ltd., Shenzhen 518031, China; hechenwei@cgnpc.com.cn; 2 China Ship Scientific Research Center, Wuxi 214082, China; panguangshan@163.com; 3 School of Mechanical Engineering, University of Science and Technology Beijing, Beijing 100083, China; 4 Physics Department, King Fahd University of Petroleum & Minerals, Dhahran 31261, Saudi Arabia; 5 K.A.CARE Energy Research & Innovation Center at Dhahran, Dhahran 31261, Saudi Arabia

**Keywords:** molecular dynamics simulation, intrinsic interlayers, diffusion bonding, hot-pressing process

## Abstract

We investigate the effect of the intrinsic interlayers on the diffusion assisted bonding properties of the austenitic steel (stainless steel 316L) and ferric steels (Low-carbon steel Q345R) in a hot rolling process by molecular dynamics simulations and experiment. The introduction of an intrinsic interlayer (Cr or Ni) widens the diffusion region, leading to enhancement of bonding. The thickness of the diffusion region enlarges with an increase of temperature, with an enhancement factor of 195% and 108%, for Cr and Ni interlayer, respectively, at the temperature of 1800 K. Further diffusion analysis reveals the unsymmetrical diffusion near the interface. Our experimental investigation evidenced our computation discovery.

## 1. Introduction

Bimetallic composites are important with broad applications in machinery, aerospace, power and electronics industries because of their superior properties. However, their quality and properties are significantly influenced by the microstructure and strength of the interfaces. There are some shortcomings in the interfaces of bimetallic composites, such as easy oxidation of the interface, creation and formation of brittle intermetallic at the interface and creation and propagation of micro cracks, which results in reducing remarkably the bonding strength of the interface. For example, some researches find that because the solubility of the elements of Fe, Ti, Cr and Ni will be severely limited in the solid state, brittle intermetallics like Fe_2_Ti, FeTi, Cr_2_Ti and Fe_2_Ti_4_O are formed in the interface [1,2,3,4,5]. In addition, because the alloying species have different intrinsic diffusion coefficients, the bimetals will create and propagate the micro cracks in the interface, which seriously affect the physical and mechanical properties [6,7]. Since the preparation process of hot roll-cladding plates is conducted at a high temperature, the bonding interface is easily oxidized, which results in remarkably reducing the bonding strength of the interface [8,9]. Hence, the key point of the research on the bimetal composite is how to improve its overall mechanical properties.

Studies have shown that the addition of intermediate interlayers on the interface with certain plasticities, strengths and affinities can improve the bonding properties of bimetal composite panels [10,11,12,13,14,15,16,17,18]. For example, detailed mechanical testing and metallurgical examinations were carried out to investigate the friction welding between TiNi alloy and austenitic stainless steel with an Ni interlayer [15]. The results indicate that due to the addition of the Ni interlayer the brittle intermetallic (Fe_2_Ti) will be drastically reduced and the mechanical property of the bimetal composite will be effectively improved, see Fukumoto et al. [15]. The tensile strengths of the interfaces of Nb/sapphire composites with and without Cr interlayers have been measured by a modified laser spallation experiment. The results show that the addition of a Cr interlayer will increase the tensile strength of the interface of Nb/sapphire composites, see Yuan et al. [17]. Despite extensive efforts by both experimental [19,20,21] and numerical methods [22,23,24,25,26], the effect mechanism of the intermediate interlayer on the bonding interface is still lacking. Such a mechanism is useful in the prediction and design of the bonding strength for the interface and helpful in optimizing the manufacturing process of bimetal composites.

Diffusion bonding is a solid-state bonding process with good mechanical and metallurgical bonding properties in the bonding area, so it is an attractive manufacturing method for aerospace applications. [27]. Ding et al. [28] used the vacuum solid-state diffusion method to combine copper and titanium on the CoCrFeMnNi high-entropy alloy intermediate layer, and investigated the influence of temperature on the diffusion reaction mechanism. Their research shows that the effective diffusion barrier layer of the combination of Cu/Ti dissimilar materials can use CoCrFeMnNi high-entropy alloy. Liu et al. [29] achieved good bonding between CoCrFeMnNi high entropy alloy and pure Cu at temperatures of 750~850 °C by solid-state diffusion method. The results show that Cu atoms diffuse into the high-entropy alloy lattice more easily. With the increase of temperature, the diffusion degree of the high-entropy alloy components towards the Cu side decreases in the order of Mn > Cr > Fe > Co > Ni. Negemiya et al. [30] investigated the influence of the diffusion bonding process parameters of AISI 410 martensitic stainless steel and nickel [Su 718]-based superalloy. From this study, it was found that the tensile strength and hardness of the joint manufactured at a bonding temperature of 980 °C, a bonding pressure of 16 MPa and a holding time of 75 min were 263 MPa and 450 HV.

The molecular dynamics simulation method has been used to study the mechanical properties of materials at the atomic scale by many scholars [31,32,33,34,35,36,37,38,39]. For example, the mechanical properties of a single metal under the tensile or compressive process have been extensively studied [40,41,42,43]. The atomic structure and mechanical and thermodynamic stability of the vacancy clusters of Cu under a uniaxial and volumetric tensile strain was examined by means of atomistic simulations by Peng et al. [44] and the nanoindentation on monocrystalline and nanotwinned Ta (nt-Ta) films were performed by means of molecular dynamics simulations by Huang et al. [45]. Besides single crystal, MD studies have been conducted on the interfaces of bimetallic composites in nanometer scale [46,47,48,49,50,51,52,53,54]. The interfacial tension strength was associated with pertinent characteristics of the interface structure in order to illustrate the impact of interfacial porosity and stresses acting on the slip-plane in non-glide directions on tensile strength of the interface and a model was proposed on the interface strength, see Douglas et al. [48]. They found that the interface is critical for mediating deformation mechanisms and mechanical properties. Weissmann et al. investigated the process of amorphization on the interface of the Co-Zr system where the degree of amorphization increased with the temperature [55]. Chen et al. investigated the atomic diffusion behavior in the process of explosive welding on the Cu-Al system and found that the atomic diffusion mainly occurred in the process of the explosive welding [56]. Chen et al. was the first to reveal that the dynamic evolution of the patterns of misfit dislocation in the interface appears in some specific types of the interface and loading schemes, which made great contributions to the mechanism of the dislocation nucleation and shear sliding [57].

Despite the extensive studies on crystals including single crystals and bi-crystals, there are few researches on bi-metal composite at the atomistic scale due to its complexities. It would be advantageous to study the composite of austenitic steel and ferritic for the combination advantages of corrosion-resistance, high strength and low price. In our previous work, the mechanics of the atomic diffusivity on the interface between bulk austenitic steel and carbon steel have been studied by using molecular dynamics simulations. However, the physical metallurgical mechanisms related to the effect of the intermediate interlayer are still elusive.

In this work, we aim to investigate the effect of an intermediate interlayer on the bonding of the interface between austenitic steel and carbon steel during the process under hot compression. Due to the outstanding corrosion resistance and broad applications of the 316L stainless steel including in chemicals, the petrochemical industry, marine applications, wastewater treatment, medical devices and more, the stainless steel (316L) was selected as our research material. In addition, the effect of some important elements in 316L stainless steel, such as Ni and Cr, on the interfacial bonding properties, was examined. The Fe_74_Cr_16_Ni_10_ alloy is used to model the 316L stainless steel by keeping the most important components (Fe, Cr and Ni) in proper concentration, as in our previous research. The reason to ignore the minor compositions is that their low concentration results in a trivial effect on the mechanical properties, as detailed in Supplementary Information. The pure Fe metal is used to model the carbon steel (Q345R) because the content of iron is over 98%. The intrinsic interlayers refer to the interlayers formed by the intrinsic elements—Fe, Cr and Ni here. The choice of these intrinsic interlayers is due to the convenience and the availability of the inter-atomic potentials. The focus of this study is the effect of the intrinsic intermediate interlayers of Cr, Ni and Fe on the interfacial diffusion and compression mechanism, which has been comprehensively studied using a FeCr_16_Ni_10_/Fe model.

## 2. Simulation Details

**Figure 1:**
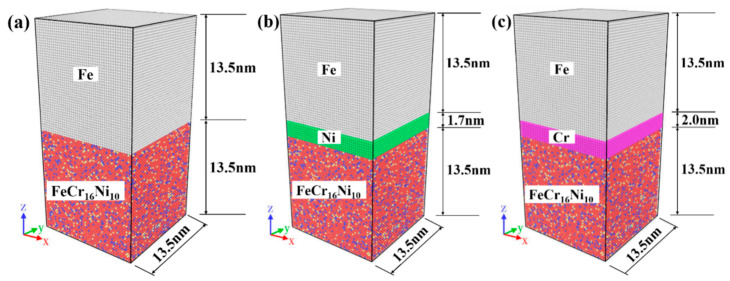
Initialization model of (**a**) Fe/FeCr_16_Ni_10_ as pristine configuration; (**b**) Fe/Ni/FeCr_16_Ni_10_ as *Ni-layer* configuration; and (**c**) Fe/Cr/FeCr_16_Ni_10_ as *Cr-layer* configuration. (The atom layer of Fe metal is on the top of model and the atom layer of FeCr_16_Ni_10_ alloy is on the bottom of model. ⬤: Fe atoms in the atom layer of Fe metal, ⬤: Fe atoms in the atom layer of FeCr_16_Ni_10_ alloy, ⬤: Cr atoms in the atom layer of FeCr_16_Ni_10_ alloy, ⬤: Ni atoms in the atom layer of FeCr_16_Ni_10_ alloy, ⬤: Ni atoms in the Ni interlayer, ⬤: Cr atoms in the Cr interlayer).

Molecular dynamics simulation was used to study the effect of the intrinsic interlayer on the diffusion bonding of the austenitic steel and ferric steel. The atomic interactions are formulated using the empirical embedded atom method (EAM) potential [58,59]. The initial model of FeCr_16_Ni_10_/Fe composite is shown in Figure 1a. The models of both Fe metal and FeCr_16_Ni_10_ alloy are put together inside a simulation box whose dimension is 13.5(*X*) × 13.5(*Y*) × 27(*Z*) nm^3^. The contact surface between Fe metal and FeCr_16_Ni_10_ alloy is the plane (0 0 1). There are 355,023 atoms in the model of the FeCr_16_Ni_10_/Fe composite, 172,773 atoms in the areas of Fe metal and 182,250 atoms in the areas of FeCr_16_Ni_10_ alloy. The initial model of Fe/Ni/FeCr_16_Ni_10_ composite is shown in Figure 1b. The Ni interlayer is put inside a simulation box whose dimension is 13.5(*X*) × 13.5(*Y*) × 1.7(*Z*) nm^3^, which contains 25,920 atoms. The initial model of Fe/Cr/FeCr_16_Ni_10_ is shown in Figure 1c. The Cr interlayer which contains 28,350 atoms is put inside a simulation box whose dimensions are 13.5(*X*) × 13.5(*Y*) × 13.5(*Z*) nm^3^. All molecular dynamics simulations are carried out by the Large-scale Atomic/Molecular Massively Parallel Simulator (LAMMPS) [60] and post-processing the atomistic data obtained from MD simulation is performed by the Open Visualization Tool (OVITO) [61].

The models along the *x* and *y* directions set the periodic boundary conditions. The velocity-Verlet algorithm is implemented to calculate the motion equation of the atoms in the simulation system with a constant time step (1 × 10^−15^ s) [62]. Before loading compression, the systems are relaxed sufficiently using a Nose-Hoover thermostat and a Nose/Hoover pressure barostat to ensure the combination of the FeCr_16_Ni_10_ alloy and Fe metal at high temperature [63,64]. The systems are kept at a pressure of zero bar and a constant temperature (T = 1500 K) for 100 ps. After relaxation, compression along the z direction is subsequently applied to the models at a temperature (1500 K) and a strain rate (3 × 10^9^ s^−1^). The relatively short time scale and the insufficient geometric scale are the limitations of molecular dynamics. Therefore, compared with actual experiments, the strain rate obtained by molecular dynamics is much higher. Since molecular dynamics is explained from the micro and nano scale, it is necessary to simplify the unimportant parameters in the experimental process. Therefore, diffusion can also be studied at high strain rates. An isobaric-isothermal (NPT) ensemble is used to keep the pressure of zero in the directions of *x* and *y* during the uniaxial compression. Twelve groups of simulations have been performed to manifest the mechanism on how the intrinsic interlayer affects the process of compression and interfacial diffusion. These models applied uniaxial compression at a constant strain rate (3 × 10^9^ s^−1^) with four temperatures (1500 K, 1600 K, 1700 K and 1800 K), which were selected because of the compromise of high diffusivity and melting point. Only if the temperature reaches adequate levels between 0.6–0.8T_m_ (where T_m_ represents the melting points of the materials involved) [18,65], can the atoms of the materials on both sides diffuse each other. Since the melting point of Fe metal and FeCr_16_Ni_10_ alloy are 1868 K and 2123 K respectively [66], the temperature levels 1500 K, 1600 K, 1700 K and 1800 K are selected in this work.

## 3. Results and Discussion

### 3.1. Stress-Strain Relationship

**Figure 2:**
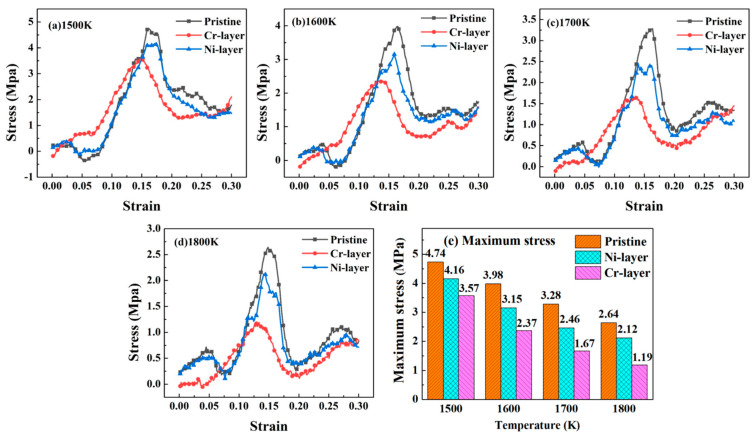
Stress-strain curves of the FeCr_16_Ni_10_/Fe composites with different intrinsic interlayer at various temperature: (**a**) 1500 K; (**b**) 1600 K; (**c**) 1700 K; (**d**) 1800 K, (**e**) The maximum stress at four temperature.

To investigate the effect of the intrinsic interlayer on the hot rolling process, the FeCr_16_Ni_10_/Fe models with and without the Ni layer (Cr layer) were conducted to simulate the hot rolling process. The stress-strain curves obtained by simulating a hot rolling process of FeCr_16_Ni_10_/Fe composites with different intrinsic interlayers at the temperatures of 1500 K, 1600 K, 1700 K and 1800 K are illustrated in Figure 2. Since the coherent interface between the FeCr_16_Ni_10_ alloy and Fe metal will lead to the lattice mismatch [67,68,69], all stresses fluctuate around zero at the initial stage. Then the further plastic deformation will require a greater compressive stress. The stress-strain curves show a drastic rise as the strain reaches a certain value in the cases (i.e., pristine, Ni-layer and Cr-layer) at different temperature ranges. After the value of stress increases to the maximum, the stress drops rapidly. Then, the interface exhibits the progressive plastic deformation in which the flow stress gradually decreases with increasing strain. According to three-stage theory on the compounding process of the hot rolled sheet, the rough surface deforms under stress loading, which increases the contact area and then two metals in contact with each other produce a coordinated plastic deformation. The bonding difficulty of a bimetallic clad plate is measured by its maximum stress during the deformation process [70].

At the temperature of 1500 K, the maximum stress of the FeCr_16_Ni_10_/Fe composite is 4.74 MPa when there is no intrinsic interlayer, namely pristine configuration. The maximum stress of the FeCr_16_Ni_10_/Fe composite reduces by 12.2% when the Ni intrinsic interlayer is present, namely Ni-layer configuration. Meanwhile, the maximum stress of the FeCr_16_Ni_10_/Fe composite reduces by 24.7% when the Cr intrinsic interlayer is present, namely Cr-layer configuration. At the temperature of 1600 K, compared with the FeCr_16_Ni_10_/Fe composite without intrinsic interlayer, the maximum stress of the FeCr_16_Ni_10_/Fe composite reduces by 20.7% with Ni-layer configuration. Meanwhile, the maximum stress of the FeCr_16_Ni_10_/Fe composite reduces by 40.3% with Cr-layer configuration. At the temperature of 1700 K, compared with the FeCr_16_Ni_10_/Fe composite without intrinsic interlayer, the maximum stresses of the FeCr_16_Ni_10_/Fe composite with the Ni-layer and Cr-layer reduce by 24.8% and 48.9%, respectively. At the temperature of 1800 K, compared with the FeCr_16_Ni_10_/Fe composite without intrinsic interlayer, the maximum stresses of the FeCr_16_Ni_10_/Fe composite with the Ni-layer and Cr-layer reduce by 29.7% and 54.9%, respectively. Obviously, the addition of the intrinsic interlayer reduces the maximum stress of the FeCr_16_Ni_10_/Fe composite on the hot rolling process and the addition of the Cr layer is more obvious than the addition of the Ni layer. So, the addition of the intrinsic interlayer can reduce the stress of bonding of the austenitic/ferric steels and is conducive to the hot rolling process of the bimetallic composites.

### 3.2. Diffusion Bonding of the FeCr_16_Ni_10_/Fe Models during the Hot Rolling

**Figure 3:**
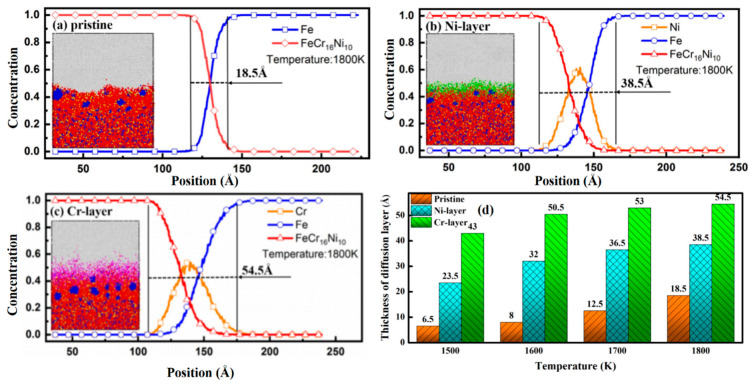
Atomic concentration distributions of FeCr_16_Ni_10_/Fe composites with different intrinsic interlayers along the *Z* direction at 1800 K with (**a**) pristine configuration; (**b**) Ni-layer; (**c**) Cr-layer and (**d**) the thicknesses of the diffusion region.

Only if the atomic concentrations of both FeCr_16_Ni_10_ alloy and Fe metal along the compression direction are more than 5%, the region of the model of the FeCr_16_Ni_10_/Fe composite involved is defined as the diffusion region [56,65]. The atomic diffusion near the interfaces of the FeCr_16_Ni_10_/Fe composite during the hot rolling process has been observed and the atomic concentration distributions of the FeCr_16_Ni_10_/Fe composite with different intrinsic interlayers along the *Z* direction at the temperature of 1800 K are illustrated in Figure 3. The atomic concentration distributions at the temperatures 1500 K, 1600 K and 1700 K are also illustrated, which are detailed in Supplementary Information. In addition, the snapshots of the model at a strain of 0.3 are also shown. The thickness of the diffusion region of the FeCr_16_Ni_10_/Fe composite without the intrinsic interlayer is 18.5 Å. However, the thickness increases by 108.1% when the Ni interlayer is added. Obviously, the Ni interlayer promotes the diffusion and combination of FeCr_16_Ni_10_ alloy and Fe metal. And good agreement was obtained when comparing our simulations with previous works. For example, the tungsten/ferric composite with a nickel interlayer which is applied to nuclear components is prepared by a diffusion bonding process and the result indicates a good diffusion bonding was obtained between the interfaces of both tungsten/nickel and nickel/ferritic by metallographic analysis [71]. It has also been observed from Figure 3b. that the atoms in the Ni intrinsic interlayer diffuse farther towards the layer of Fe metal than towards the layer of FeCr_16_Ni_10_ alloy, which indicates that atoms in the Ni intrinsic interlayer are more likely to diffuse into the layer of Fe metal. That is because the concentration gradient of Ni between the Ni intrinsic interlayer and the layer of Fe metal is higher than between the Ni interlayer and the layer of FeCr_16_Ni_10_ alloy.

The thickness of the diffusion region increases by 194.6% when the Cr intrinsic interlayer is present, as can be seen from Figure 3c. Obviously, the Cr intrinsic interlayer also promotes the diffusion and adhesion of FeCr_16_Ni_10_ alloy and Fe metal. Due to the higher atomic concentration of the Cr intrinsic interlayer, a higher concentration gradient between the Cr intrinsic interlayer and the FeCr_16_Ni_10_ alloy (the Fe metal) will appear, which causes the thickness of the diffusion region between FeCr_16_Ni_10_ alloy and Fe metal to increase. Meanwhile, the effect of the Cr intrinsic interlayer on the diffusion and adhesion of FeCr_16_Ni_10_ alloy and Fe metal is much better than the Ni intrinsic interlayer. This is because the diffusion coefficient of the element of Cr is larger than the element Ni and the binding energies of the element of Cr are smaller than the element of Ni. In previous studies, the diffusivity of Cr was found to be 6.9745 × 10^−10^ m^2^s^−1^ and the diffusivity of Ni was 6.5144 × 10^−10^ m^2^s^−1^ [62]. In addition, similar to the Ni intrinsic interlayer, the atoms in the Cr intrinsic interlayer diffuse farther towards the layer of Fe metal than towards the layer of FeCr_16_Ni_10_ alloy, which indicates that atoms in Cr intrinsic interlayer are more likely to diffuse into the layer of Fe metal. That is because the concentration gradient of Cr between the Cr intrinsic interlayer and Fe metal is also higher than between the Cr intrinsic interlayer and FeCr_16_Ni_10_ alloy.

All in all, the addition of the pure Ni or Cr intrinsic interlayer could supply faster diffusion of compositional element and enhance the adhesion of the interface. The effect of the Cr intrinsic interlayer is much better than the Ni intrinsic interlayer because the binding energies of the element of Cr is smaller than that of the element of Ni and the diffusion coefficient of the element of Cr is larger than the element of Ni [72].

The thicknesses of the diffusion region of FeCr_16_Ni_10_/Fe composite with different intrinsic interlayers at the temperatures of 1500 K, 1600 K, 1700 K and 1800 K are illustrated in Figure 3d. The thickness of the diffusion region of FeCr_16_Ni_10_/Fe composite with pristine configuration is 6.5 Å at the temperature of 1500 K. The thicknesses of the diffusion region of FeCr_16_Ni_10_/Fe composite with the addition of the Ni-layer and Cr-layer increase by 261.5% and 561.5%, respectively. The thickness of the diffusion region of FeCr_16_Ni_10_/Fe composite with pristine configuration is 8 Å at a temperature of 1600 K. The thicknesses of the diffusion region of FeCr_16_Ni_10_/Fe composite with the addition of Ni-layer and Cr-layer increase by 300% and 531.3%, respectively. The thickness of the diffusion region of FeCr_16_Ni_10_/Fe composite with pristine configuration is 12.5 Å at the temperature of 1700 K. The thicknesses of the diffusion region of FeCr_16_Ni_10_/Fe composite with the addition of Ni-layer and Cr-layer increase by 192% and 324%, respectively. The thickness of the diffusion region of FeCr_16_Ni_10_/Fe composite with pristine configuration is 18.5 Å at the temperature of 1800 K. The thicknesses of the diffusion region of FeCr_16_Ni_10_/Fe composite with the addition of Ni-layer and Cr-layer increase by 108% and 195%, respectively. Obviously, the addition of the Ni or the Cr intrinsic interlayer induces a thicker diffusion region, which leads to enhancement of adhesion. For such enhancement, the Cr intrinsic interlayer has much better performance than the Ni intrinsic interlayer.

### 3.3. Amorphization Degree of the Interface of the FeCr_16_Ni_10_/Fe Composite

The radius distribution function (RDF) might be used to evaluate the influence of the intrinsic interlayer on the amorphization degree of the interface, by which the atomic spatial distributions and structures near the interface can be characterized. First of all, the region where the diffusion region is thickest is defined as the interface. Secondly the amorphization degree of the interface of FeCr_16_Ni_10_/Fe composite with different intrinsic interlayers can be compared by the width and height of the peaks of the RDF curves in the interface. And the broader and lower peak appears, the higher degree of amorphization will be.

**Figure 4:**
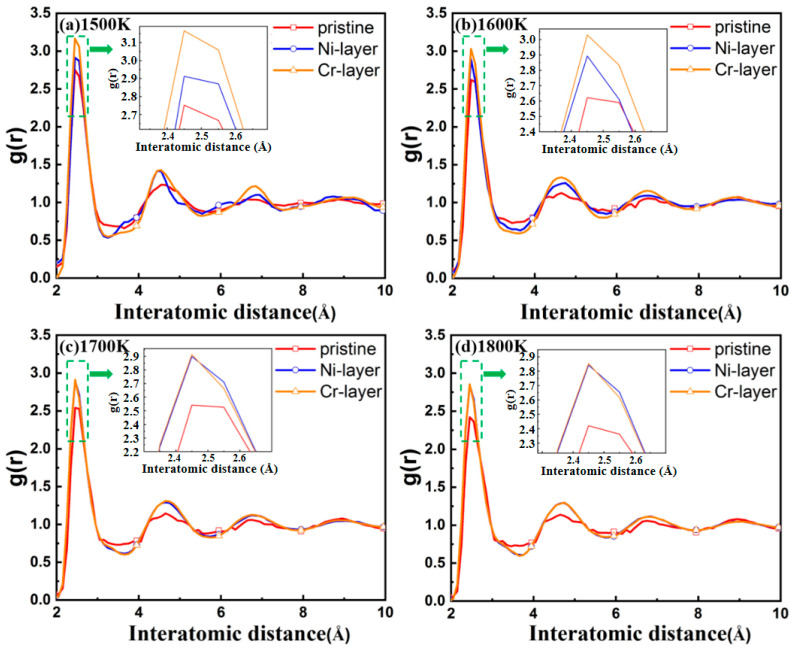
Radius distribution functions of FeCr_16_Ni_10_/Fe composites with the different intrinsic interlayers on the interface at different temperatures: (**a**) 1500 K; (**b**) 1600 K; (**c**) 1700 K; (**d**) 1800 K. The insert images show a first narrower peak appears at the interatomic distance of 2.61 Å.

The RDFs of the interface of FeCr_16_Ni_10_/Fe composite with different intrinsic interlayers at various temperature have been examined, as shown in Figure 4. Similar to liquid, the RDF curves of the interfaces with no intrinsic interlayer all tend rapidly to be united after the second neighbor peak, which indicates short-range order and long-range disorder, from which we can conclude that an amorphous structure has appeared in the interface. This indicates a good agreement between the RDF curves and the previous visualization of atomic structure of the interfaces with no intrinsic interlayer (in Figure 3). However, the RDF curves of the interfaces with the addition of the Ni or the Cr intrinsic interlayer show sharper peaks, which indicates that a more crystalline structure appears. In short, all the above show that the interface structure changes from an amorphous state to a crystalline state when the intrinsic interlayer is present.

## 4. Experimental Analysis

To validate of our molecular dynamics simulations, we have compared our results to the experimental investigations. Due to the limitation of resources and difficulties of high temperature experiments, including temperature control and corrosion control, the experiments on the thin interlayers are very limited during the hot-rolling process. We only compared and analyze the hot-rolling process with and without Ni interlayers to check if the interlayer is assisting the bonding.

For the specimen with FeCr_16_Ni_10_ alloy and Fe metal layers, the stainless clad plates with the Ni intrinsic interlayer and without the Ni intrinsic interlayer were prepared by the hot rolling process. The microstructure and the diffusion of elements in the interface of the stainless clad plate was studied by using an optical microscope (OM, ZEISS, Jena, Germany) and energy dispersive spectrum (EDS, Thermo Fisher Scientific, Waltham, MA, USA). The experimental materials were 316L stainless steel (composite) and Q345 low carbon steel (substrate). The size of 316L stainless steel was 300 mm × 300 mm × 10 mm, and the size of Q345 low carbon steel was 300 mm × 300 mm × 90 mm. The original thickness of the Ni layer was 0.1 mm. The experiment consisted of four steps. First, the surface of the 316L steel and Q345 steel was machined to a certain roughness. Second, the surrounding area was welded and evacuated, and the degree of vacuum was 0.1 Pa. Third, the sealed composite slab was heated to 1473 K for 2 h and then at this temperature rolled on a two-roll hot rolling mill. The finishing temperature was controlled between 1223 K and 1323 K. Finally, the hot-rolled sample was polished, and then the carbon steel layer was etched with nital 4. The distribution of major elements on the two sides of the composite interface were analyzed using an EDS. The microscopic characteristics of the interface were observed using a 200 MAT optical microscope.

In contrast to the reference [73], the macro interface is not a straight line, but a slightly curved curve. The structure of the Q345 steel away from the interface is ferrite and pearlite, and the structure of the Q345 steel from the interface of 150–200 μm is ferrite, which indicates that there is a certain degree of decarburization in this area. Since the carbon in the Q345 steel is easily diffused into the 316L stainless steel under high temperature and deformation conditions, the decarburization occurs on the side of low carbon steel near the composite interface. The experiment of the hot-rolling process with the Ni intrinsic interlayer shows that the decarburization zone on the side of the Q345 steel near the interface is very narrow and less than 50 μm, which indicates that the diffusion of the element of C can be significantly prevented by the presence of the Ni intrinsic interlayer [73].

The distributions of the elements like Cr and Ni on the two sides of the interface of the composite without the Ni intrinsic interlayer were analyzed by using the EDS, as studied in the reference [73]. The elements such as Fe, Cr and Ni on both sides of the interface vary greatly. The elements such as Ni and Cr in the stainless steel (316L) diffuse through the original interface to the low-carbon steel (Q345). The diffusion distance of Cr is about 20 μm and the diffusion distance of Ni is about 8 μm. On one hand, the concentration gradient of the element of Ni on both sides of the interface is lower than the element of Cr and on the other, this is because the diffusion coefficient of the element of Ni is lower than the element of Cr. The distribution of elements such as Cr and Ni on the two sides of the interface of the composite with the Ni intrinsic layer was also analyzed by using the EDS. Compared to the reference [73], the Cr element in the stainless steel (316L) diffuses into the Ni intrinsic interlayer, which causes a decrease in the Cr content. And the elements of Fe in the stainless steel (316L) and the low-carbon steel (Q345R) diffuse into the Ni intrinsic interlayer, while the elements of Ni in the Ni intrinsic interlayer diffuse into both sides of the interface. The diffusion distances of the element of Ni both between the Ni intrinsic interlayer and the layer of the low-carbon steel (Q345R) and between the Ni intrinsic interlayer and the layer of the stainless steel (316L) are 25 μm and 20 μm, respectively. The reason the diffusion distance of the element of Ni between Ni intrinsic interlayer and the low-carbon steel (Q345R) is longer than between the Ni intrinsic interlayer and the layer of stainless steel (316L) is that the concentration gradient of the element of Ni between the Ni intrinsic interlayer and the low-carbon steel is bigger than between the Ni intrinsic interlayer and the stainless steel. This is consistent with the previous visualization of atomic structure in Figure 3b. The Ni elements in the Ni intrinsic interlayer asymmetrically diffuse to both sides of the interface and proceed with diffusion. This results in the concentration gradients between the Ni intrinsic interlayer and both sides reducing and gradually slowing down. Compared to the diffusion region of the hot rolled sheets with and without the Ni intrinsic interlayer by the EDS analysis, the thickness increases when the Ni intrinsic interlayer is present. Due to the higher atomic concentration of the Ni intrinsic interlayer, a higher concentration gradient between the Ni intermediate layer and the stainless steel (316L) (the low-carbon steel (Q345R)) makes the thickness of the diffusion region increase.

## 5. Conclusions

To study the influence of the intrinsic interlayers on the diffusion assisted bonding properties of the austenitic steel and ferric steels in a hot rolling process, the molecular dynamics simulation and experiment have been used in this work. Our research reveals the effect of the intrinsic interlayer on the bonding process in order to provide a theoretical basis for optimizing the manufacturing process of the hot rolled sheet. The main conclusions are as follows:

(1) Through molecular dynamics simulation, we found that the addition of the intrinsic interlayer can reduce the maximum stress between the austenitic steel and carbon steel during the hot rolling process and is conducive to the bonding of the austenitic steel and carbon steel. In addition, the intrinsic inter-layer improves the adhesion of the austenitic steel and carbon steel and makes the diffusion region thicker. The effect of the Cr intrinsic interlayer is superior to the Ni intrinsic interlayer.
(2) Experimental investigation shows that the thickness of the diffusion region between the stainless steel (316L) and the low-carbon steel (Q345R) increases when the intrinsic interlayer is present. And the atoms in intrinsic interlayer diffuse farther towards the carbon steel than the austenitic steel, which indicates that atoms in the intrinsic interlayer are more likely to diffuse into the carbon steel.

## Data Availability

The data presented in this study are available on request from the corresponding author.

## Supplementary Materials

The following are available online at https://www.mdpi.com/article/10.3390/ma14092416/s1, Figure S1. Atomic concentration distributions of the FeCr_16_Ni_10_/Fe composite with different intrinsic interlayers along the *Z* direction at 1500K with (**a**) pristine configuration; (**b**) Ni-layer; (**c**) Cr-layer; Figure S2. Atomic concentration distributions of FeCr_16_Ni_10_/Fe composites with different intrinsic interlayers along the *Z* direction at 1600K with (**a**) pristine configuration; (**b**) Ni-layer; (**c**) Cr-layer; Figure S3. Atomic concentration distributions of FeCr_16_Ni_10_/Fe composites with different intrinsic interlayers along the *Z* direction at 1700K with (**a**) pristine configuration; (**b**) Ni-layer; (**c**) Cr-layer; Table S1. The Elements Composition of Materials (%).
