# Supplementary material for: Enhancement of Diffusion Assisted Bonding of the Bimetal Composite of Austenitic/Ferric Steels via Intrinsic Interlayers
Source: Materials (Basel). 2021 May 6;14(9):2416. doi: 10.3390/ma14092416 (PMC8125153; doi:10.3390/ma14092416)
Supplement: Supplementary file 1 [file materials-14-02416-s001.zip › materials-1169285-supplementary.pdf]

materials

Supplementary Information: Enhancement of Diffusion Assisted Bonding of the Bimetal Composite of Austenitic/Ferric Steels via Intrinsic Interlayers

Chenwei He, Guangshan Pan, Lu Xie and Qing Peng

1. MD Model of $FeCr_{16}Ni_{10}$/Fe Alloy

Two kinds of alloys including the stainless steel (316L) and the carbon steel (Q345R), were intentionally used to the hot rolling simulation due to their importance as structural materials in industry. As can be seen from Table S1, various kinds of elements (>9) are contained in the stainless steel (316L) and the carbon steel (Q345R), which leads to a great challenge in modeling at the atomic scale. To overcome the difficulties, those prominent components whose concentrations are more than 10% were only considered in modeling. The elements with low concentration in the stainless steel (316L) and carbon steel (Q345R) are generally believed to be less important for the interface morphology and are ignored in this study. Therefore, the carbon steel (Q345R) was considered as pure Fe without any other elements. That is because the element of Fe is predominated with over 98%. The stainless steel (316L) was simplified to the alloy only containing the elements of Fe, Cr and Ni, where the mass fraction of the elements of Cr and Ni are 16.03% and 10.03%, respectively, as shown in Table S1. Combining the two parts forming the interface, the model of $FeCr_{16}Ni_{10}$/Fe composite was applied in this study.

Table S1. The Elements Composition of Materials (%).

Materials	C	Si	Mn	P	S	Cr	Ni	Mo	Al	Ti
Q345R	0.17	0.27	1.40	0.011	0.007	0.023	0.02	<0.01	<0.01	<0.01
SUS316L	0.017	0.42	1.26	0.031	0.003	16.03	10.03	1.95		

2. Diffusion Bonding of the $FeCr_{16}Ni_{10}$/Fe Models during the Hot Rolling

The atomic concentration distributions of the hot rolled sheets with different intrinsic interlayers along the *Z* direction at the temperature of 1500K are illustrated in Figure S1. The snapshots of the model at the strain of 0.3 are also shown. The thickness of the diffusion layer of the $FeCr_{16}Ni_{10}$/Fe composite without the intrinsic interlayer is 6.5Å. However the thickness increases by 261.5% when the Ni intrinsic interlayer is present. Meanwhile, the thickness increases by 561.5% when the Cr intrinsic interlayer is added. Obviously, the intrinsic interlayer promotes the diffusion and combination of $FeCr_{16}Ni_{10}$ alloy and Fe metal. The effect of the Cr intrinsic interlayer on the diffusion and combination of $FeCr_{16}Ni_{10}$ alloy and Fe metal is much better than the Ni intrinsic interlayer.

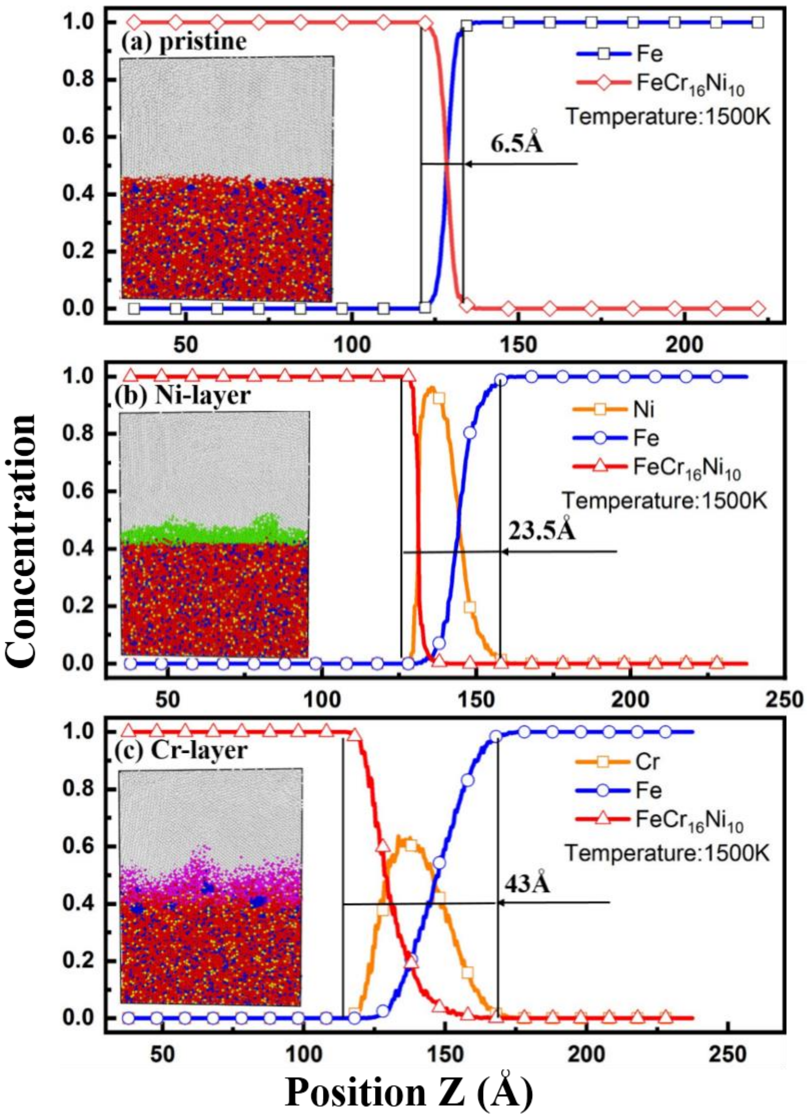

Figure S1. Atomic concentration distributions of the $FeCr_{16}Ni_{10}$/Fe composite with different intrinsic interlayers along the *Z* direction at 1500K with (**a**) pristine configuration; (**b**) Ni-layer; (**c**) Cr-layer.

The atomic concentration distributions of the $FeCr_{16}Ni_{10}$/Fe composites with different intrinsic interlayers along the *Z* direction at the temperature of 1600K are illustrated in Figure S2. The snapshots of the model at the strain of 0.3 are also shown. The thickness of the diffusion layer of the $FeCr_{16}Ni_{10}$/Fe composites without the intrinsic interlayer is 8Å. However the thickness increases by 300% when the Ni intrinsic interlayer is present. Meanwhile, the thickness increases by 531.3% when the Cr intrinsic interlayer is added. Obviously, the intrinsic interlayer promotes the diffusion and combination of $FeCr_{16}Ni_{10}$ alloy and Fe metal. The effect of the Cr intrinsic interlayer on the diffusion and combination of $FeCr_{16}Ni_{10}$ alloy and Fe metal is much better than the Ni intrinsic interlayer.

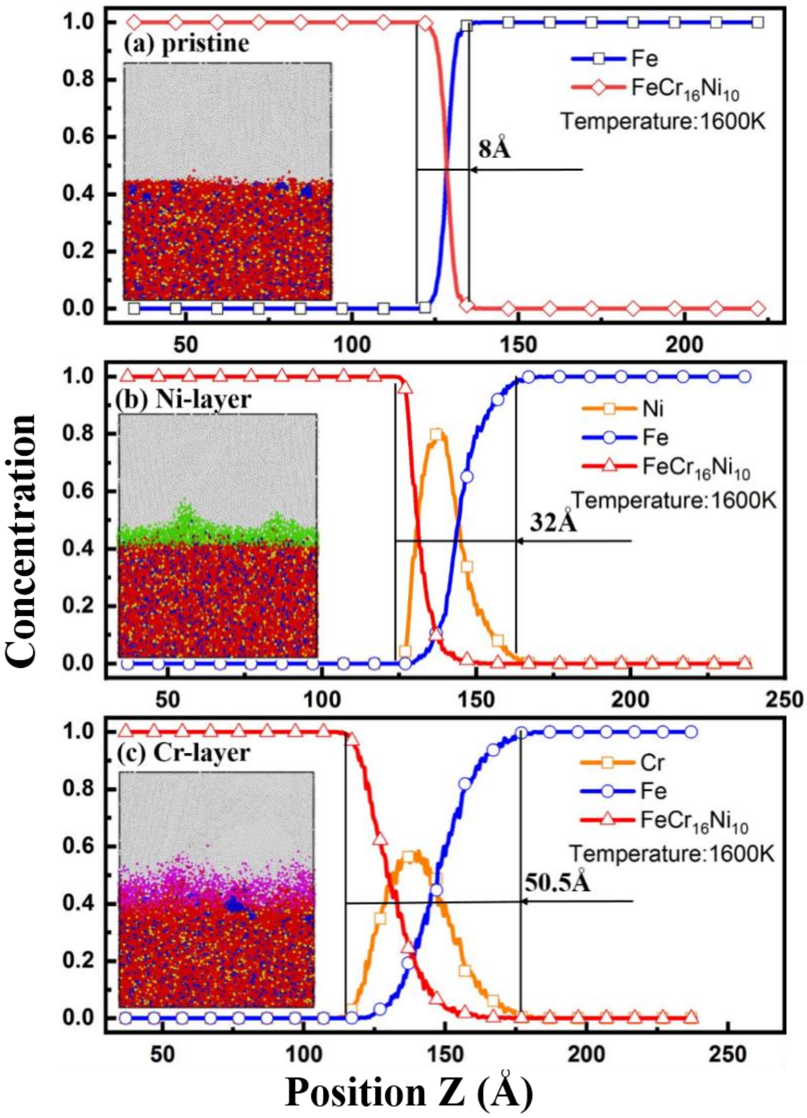

Figure S2. Atomic concentration distributions of $FeCr_{16}Ni_{10}$/Fe composites with different intrinsic interlayers along the *Z* direction at 1600K with (**a**) pristine configuration; (**b**) Ni-layer; (**c**) Cr-layer.

The atomic concentration distributions of $FeCr_{16}Ni_{10}$/Fe composites with different intrinsic interlayers along the *Z* direction at the temperature of 1700K are illustrated in Figure S3. The snapshots of the model at the strain of 0.3 are also shown. The thickness of the diffusion layer of $FeCr_{16}Ni_{10}$/Fe composites without the intrinsic interlayer is 12.5Å. However the thickness increases by 192% when the Ni intrinsic interlayer is present. Meanwhile, the thickness increases by 324% when the Cr intrinsic interlayer is added. Obviously, the intrinsic interlayer promotes the diffusion and combination of $FeCr_{16}Ni_{10}$ alloy and Fe metal. The effect of the Cr intrinsic interlayer on the diffusion and combination of $FeCr_{16}Ni_{10}$ alloy and Fe metal is much better than the Ni intrinsic interlayer.

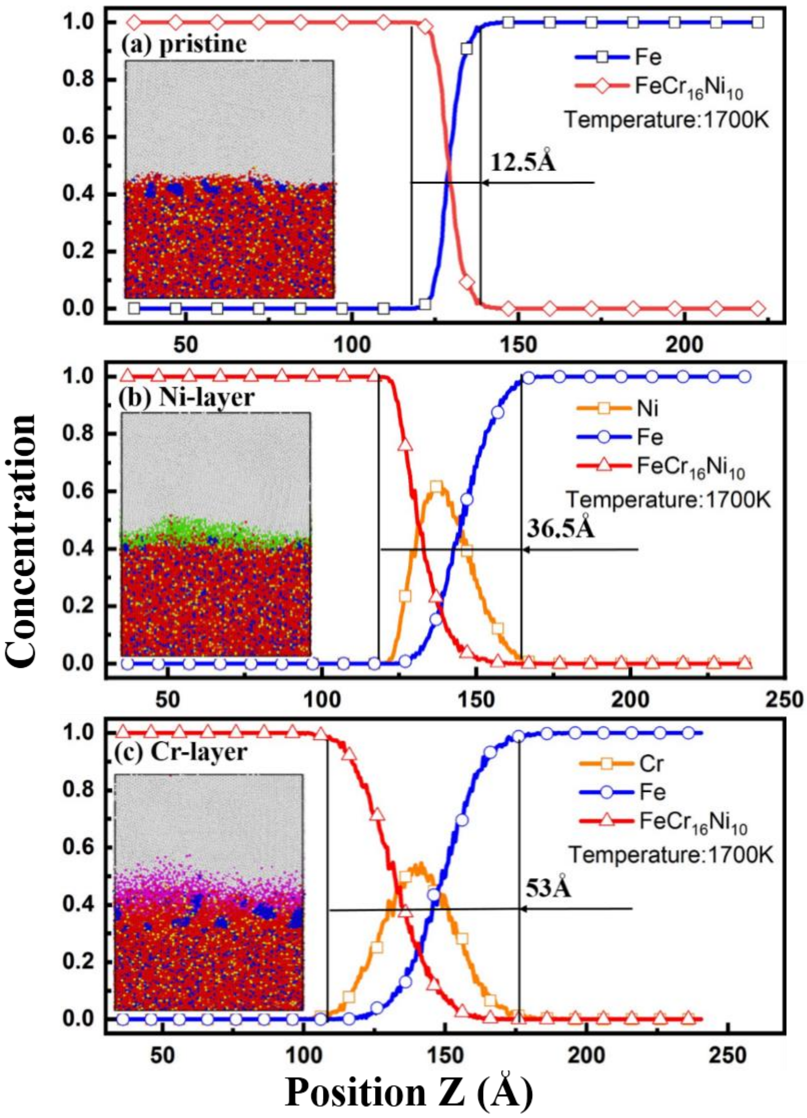

Figure S3. Atomic concentration distributions of $FeCr_{16}Ni_{10}$/Fe composites with different intrinsic interlayers along the *Z* direction at 1700K with (**a**) pristine configuration; (**b**) Ni-layer; (**c**) Cr-layer.
